# Chemical Genomics Approach Leads to the Identification of Hesperadin, an Aurora B Kinase Inhibitor, as a Broad-Spectrum Influenza Antiviral

**DOI:** 10.3390/ijms18091929

**Published:** 2017-09-08

**Authors:** Yanmei Hu, Jiantao Zhang, Rami Musharrafieh, Raymond Hau, Chunlong Ma, Jun Wang

**Affiliations:** 1 Department of Pharmacology and Toxicology, College of Pharmacy, University of Arizona, Tucson, AZ 85721, USA; yanmeihu@email.arizona.edu (Y.H.); jiantao@pharmacy.arizona.edu (J.Z.); rayhau@email.arizona.edu (R.H.); 2 Department of Chemistry and Biochemistry, University of Arizona, Tucson, AZ 85721, USA; ramim@email.arizona.edu; 3 BIO5 Institute, University of Arizona, Tucson, AZ 85721, USA; cma@pharmacy.arizona.edu

**Keywords:** influenza, broad-spectrum antiviral, host-targeting antiviral, hesperadin, drug resistance, combination therapy, aurora kinase

## Abstract

Influenza viruses are respiratory pathogens that are responsible for annual influenza epidemics and sporadic influenza pandemics. Oseltamivir (Tamiflu^®^) is currently the only FDA-approved oral drug that is available for the prevention and treatment of influenza virus infection. However, its narrow therapeutic window, coupled with the increasing incidence of drug resistance, calls for the next generation of influenza antivirals. In this study, we discovered hesperadin, an aurora B kinase inhibitor, as a broad-spectrum influenza antiviral through forward chemical genomics screening. Hesperadin inhibits multiple human clinical isolates of influenza A and B viruses with single to submicromolar efficacy, including oseltamivir-resistant strains. Mechanistic studies revealed that hesperadin inhibits the early stage of viral replication by delaying the nuclear entry of viral ribonucleoprotein complex, thereby inhibiting viral RNA transcription and translation as well as viral protein synthesis. Moreover, a combination of hesperadin with oseltamivir shows synergistic antiviral activity, therefore hesperadin can be used either alone to treat infections by oseltamivir-resistant influenza viruses or used in combination with oseltamivir to delay resistance evolution among oseltamivir-sensitive strains. In summary, the discovery of hesperadin as a broad-spectrum influenza antiviral offers an alternative to combat future influenza epidemics and pandemics.

## 1. Introduction

Influenza viruses are RNA viruses that belong to the *Orthomyxoviridae* family [1]. There are four types of influenza viruses, A, B, C, and D, among which influenza A and B viruses are responsible for annual influenza epidemics, and influenza A viruses also account for sporadic influenza pandemics. Influenza A viruses are further classified as different subtypes according to their surface antigens, hemagglutinin (HA) and neuraminidase (NA) [2]. There are 18 subtypes of HA and 11 subtypes of NA, and they can theoretically undergo all possible combinations. The subtypes that are circulating among humans are predominantly H1N1 and H3N2, and H2N2 was also prevalent in the past. Influenza B viruses are classified as Victoria and Yamagata lineages. Influenza A and B viruses co-circulate in each influenza season; however, the constitution and ratio of influenza A and B viruses vary in each influenza season, which presents a great challenge in devising influenza vaccines [3].

Infection in healthy immunocompetent adults with seasonal influenza viruses is generally self-limited and does not lead to critical illness. However, children younger than five years old, seniors older than 65 years old, immunocompromised patients, and people with chronic diseases such as asthma, chronic obstructive pulmonary disease and diabetes are at high risk for complications from influenza and need early intervention, according to the Centers for Disease Control and Prevention (CDC) [4]. Moreover, human infection with avian influenza viruses such as H5N1 and H7N9 has a mortality rate of greater than 35%, regardless of the age group and health status [5,6].

There are two classes of FDA-approved influenza antivirals, M2 channel blockers (amantadine and rimantadine) [7] and neuraminidase inhibitors (oseltamivir, zanamivir, and peramivir) [8]. More than 95% of the current circulating influenza A viruses are resistant to adamantanes [9], prompting CDC to not recommend the use of these drugs. Although the majority of the current circulating influenza strains are sensitive to oseltamivir, the number of oseltamivir-resistant strains continues to increase [10,11]. More alarmingly, the 2007–2008 seasonal H1N1 influenza virus circulating in North America was completely resistant to oseltamivir due to the H275Y mutation [10], suggesting oseltamivir-resistant strains have gained transmission fitness. Although the oseltamivir-resistant seasonal H1N1 strain was overtaken by the oseltamivir-sensitive pandemic H1N1 virus in 2009, it is unpredictable when oseltamivir-resistant strains might re-emerge in the near future. Moreover, oseltamivir has a narrow therapeutic window and has to be administered within 48 h after the onset of the symptoms [12]. In addition, oseltamivir has limited efficacy in treating critically ill patients [13]. Therefore, a next generation of influenza antivirals with a novel mechanism of action is clearly needed [14]. To address this unmet medical need, we are interested in targeting host factors that are essential for influenza virus replication as a novel approach to discover antiviral drugs. Advantages of targeting host factors include, but not limited to, a broader antiviral spectrum and higher genetic barriers to drug resistance.

**Figure 1:**
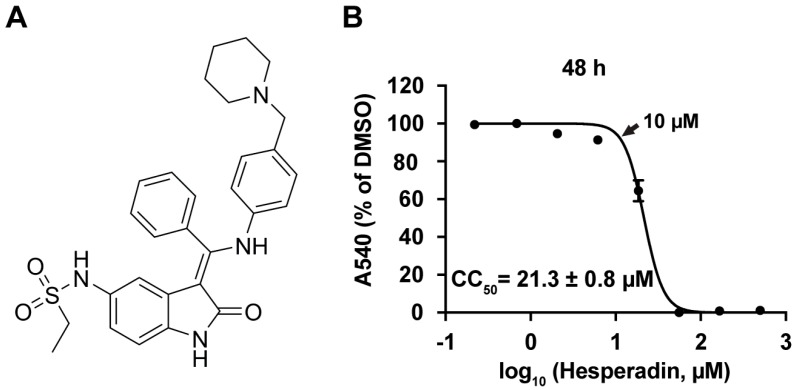
(**A**) Chemical structure of hesperadin; (**B**) Cytotoxicity of hesperadin. Serial concentrations of hesperadin were added to Madin-Darby Canine Kidney (MDCK) cells and incubated for 48 h. The cell viability was determined by neutral red up-taken assay [24]. The CC_50_ was calculated from the best-fit dose response curves with variable slope in Prism 5. The arrow indicates the highest concentration used in Figure 2. The CC_50_ value represents the average of eight repeats ± standard deviation.

Influenza viruses require host cells for viral replication. Therefore, host factors are important antiviral drug targets [15]. The host factors required for influenza virus replication have been systematically studied and a list of high profile host factors have been identified through siRNA knockdown or pulldown assays [16,17,18,19,20,21,22]. In light of this progress, burgeoning progress in pursuing host-targeting influenza antivirals has been seen in recent years, and there are currently a number of host-targeting antivirals in pre-clinic and clinic development [8,14]. In this study, we took a forward chemical genomics approach by screening a library of bioactive compounds with known biological functions against the A/WSN/33 (H1N1) virus, and identified hesperadin (Figure 1A), an aurora kinase B inhibitor, that has potent antiviral activity. Hesperadin is an ATP-competitive inhibitor of aurora B kinase with IC_50_ of 250 nM [23]. It also inhibits other kinases such as AMPK, Lck, MKK1, MAPKAP-K1, CHK1, and PHK at 1 µM drug concentration [23]. We found that hesperadin inhibits multiple human clinic isolates of influenza A and B viruses with single to submicromolar efficacy. Mechanistic studies have shown that hesperadin inhibits the early stage of viral replication post viral entry and delays the nuclear import of the viral ribonucleoprotein complex. As a result, viral RNA transcription and translation, and viral protein synthesis are inhibited. As the antiviral mechanism of hesperadin is distinct from oseltamivir, combination therapy of hesperadin with oseltamivir was found to have synergistic antiviral activity. Overall, the discovery of hesperadin as a broad-spectrum host-targeting influenza antiviral is significant as it suggests that hesperadin can be used as a second line of defense should oseltamivir fail in the event of an influenza epidemic or pandemic.

## 2. Results

### 2.1. Hesperadin Inhibits Multiple Human Clinic Isolates of Influenza A and B Viruses

**Figure 2:**
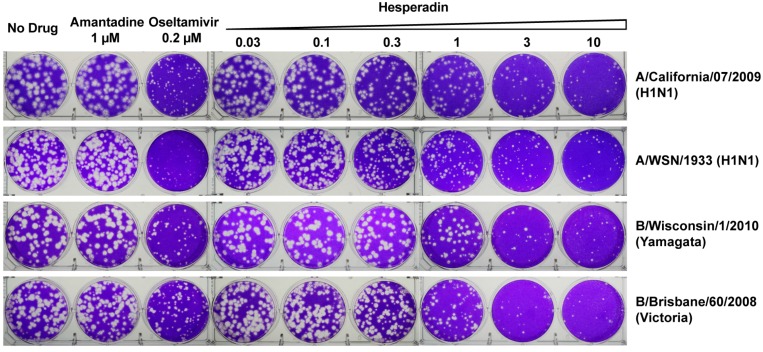
Plaque assays of hesperadin in inhibiting influenza A and B viruses. The assay was carried out with MDCK cells. Plaque areas were determined by Image J at each concentration, and the data was fit into a dose-response curve with Prism 5. The best fit EC_50_ values are shown in Table 1. The plaque assay was tested in duplicate at each drug concentration.

The antiviral efficacy of hesperidin was tested in plaque assay. To ensure the antiviral activity of hesperadin was not due to its cellular cytotoxicity, we first tested the cellular cytotoxicity of hesperadin against the Madin–Darby Canine Kidney (MDCK) cell, which is the cell line used for the plaque assay [25]. The CC_50_ value of hesperadin is 21.3 ± 0.8 µM with a 48 h incubation time (Figure 1B). As such, the highest drug concentration for hesperadin was set as 10 µM in the plaque assay, which confers minimal to no cellular cytotoxicity. It was found that hesperadin inhibits multiple human clinic isolates of influenza A and B viruses with single to submicromolar efficacy and the selectivity index (SI) ranges from 9.7 to 76.1 (Table 1), indicating hesperadin has a favorable therapeutic window. Representative plaque images were shown in Figure 2. Hesperadin was not only active against oseltamivir-sensitive strains such as A/California/07/09 (H1N1), B/Wisconsin/1/2010 (Yamagata), and B/Brisbane/60/2008 (Victoria), but also the oseltamivir and amantadine dual-resistant strains such as A/Texas/4/2009 (H1N1), A/North Carolina/39/2009 (H1N1), A/Washington/29/2009 (H1N1), A/California/02/2014 (H3N2), and A/Texas/12/2007 (H3N2) viruses. Overall, the discovery of hesperadin as a broad-spectrum influenza antiviral with similar potency against multiple types and subtypes of influenza viruses warrants its further development.

### 2.2. Hesperadin Inhibits Viral Replication in Cells Infected with a High Multiplicity of Infection (MOI)

**Figure 3:**
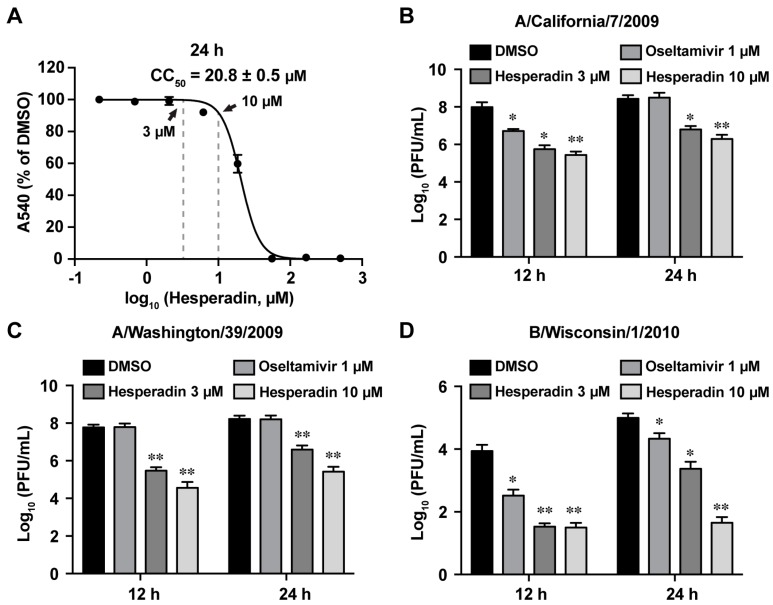
Antiviral activity of hesperadin against A/California/07/2009 (H1N1), A/Washington/39/2009 (H1N1) and B/Wisconsin/1/2010 (Yamagata) at a high multiplicity of infection (MOI) of 1. (**A**) CC_50_ of hesperadin with a 24 h incubation time in MDCK cells. The concentrations at 3 and 10 µM are indicated by arrows; the CC50 value represents the average of eight repeats ± standard deviation; MDCK cells were infected with A/California/7/2009 (H1N1) (**B**) A/Washington/39/2009 (H1N1) (**C**), or B/Wisconsin/1/2010 (**D**) at a MOI of 1, 3 or 10 µM of hesperadin was added after viral infection. Viruses from supernatant were harvested at 12 or 24 h p.i. Virus titers were determined by plaque assay as previously described [27]. 1 µM of oseltamivir carboxylate was included as a control. Asterisks indicate statistically significant difference in comparison with the DMSO control (student’s *t*-test, * *p* < 0.05, ** *p* < 0.01). The results represent the average of two repeats ± standard deviation.

To test whether hesperadin inhibits viral replication in cells that are infected with a high MOI, a condition which mimics late treatment post viral infection in the clinical setting, we evaluated the antiviral efficacy of hesperadin against three human clinic isolates of influenza viruses, A/California/7/2009 (H1N1), A/Washington/39/2009 (H1N1), and B/Wisconsin/1/2010 (Yamagata), when the cells were infected with a MOI of 1. The viral titers at the supernatant were quantified at 12 and 24 h p.i. by plaque assay. Cells were treated with either 3 or 10 µM hesperadin, both of which have minimal cellular cytotoxicity with a 24 h incubation time (Figure 3A). Oseltamivir was included as a control. For the oseltamivir-sensitive strain A/California/7/2009 (H1N1), 1 µM oseltamivir carboxylate reduced the viral titer by 1.3 log_10_ units at 12 h p.i. but had no effect at 24 h p.i. (Figure 3B). In contrast, hesperadin significantly reduced the viral titer by 1.6–2.5 log_10_ units at both 12 and 24 h p.i., and the inhibition was dose dependent with 10 µM drug treatment leading to greater viral titer reduction. For the oseltamivir-resistant strain A/Washington/39/2009 (H1N1) oseltamivir had no effect on the viral titer at both 12 and 24 h p.i. when tested at 1 µM (Figure 3C). In contrast, hesperadin showed potent antiviral activity by reducing the viral titers by 2.3 and 3.2 log_10_ units at 3 and 10 µM, respectively, at 12 h p.i. Similarly, the viral titers were reduced by 1.6 and 2.8 log_10_ units at 3 and 10 µM, respectively, at 24 h p.i. For the influenza B strain, B/Wisconsin/1/2010 (Yamagata), hesperadin reduced the viral titer by more than 2 log_10_ units at both 12 and 24 h p.i. and had a more potent antiviral efficacy than oseltamivir (Figure 3D). Collectively, our results showed that hesperadin is more efficacious in inhibiting viral replication in cells that are infected with a high MOI.

### 2.3. Hesperadin Inhibits the Early Stage of Viral Replication Post Viral Entry

**Figure 4:**
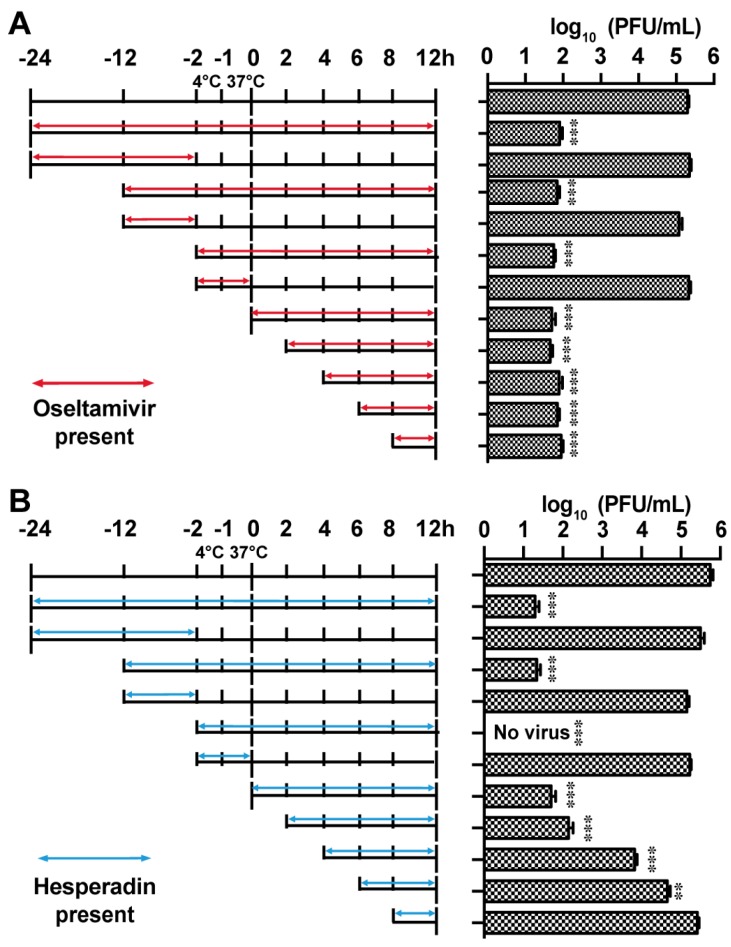
Time-of-addition experiments. MDCK cells were infected with the A/WSN/33 (H1N1) at MOI of 0.01 at −2 h time point; viruses were first incubated at 4 °C for 1 h for attachment followed by 37 °C for 1 h for viral entry. At time point 0 h, cells were washed with PBS buffer and viruses were harvested at 12 h p.i. The titer of harvested virus was determined by plaque assay. Arrows indicate the period in which (**A**) 1 µM oseltamivir carboxylate or (**B**) 3 µM hesperidin was present. Asterisks indicate statistically significant difference in comparison with the DMSO control (student’s *t*-test, ** *p* < 0.01, *** *p* < 0.001). The results represent the average of two repeats ± standard deviation.

To investigate the antiviral mechanism of action of hesperadin, we first performed the drug time-of-addition experiment (Figure 4), in which hesperadin was added at different time points either prior, during, or post viral infection and the viral titer was quantified by plaque assay at 12 h p.i. [28]. In the time-of-addition experiment, MDCK cells were infected with A/WSN/33 (H1N1) virus at a MOI of 0.01 at −2 h time point and were kept at 4 °C for 1 h to allow viral attachment. This was followed by 1 h incubation at 37 °C for viral entry. Next, the viral inoculum was removed by aspirating, followed by PBS washing, and the resulting cell culture was incubated at 37 °C for 12 h. The viral titer in the supernatant at 12 h time point was titrated by plaque assay. Oseltamivir carboxylate was included as a control (Figure 4A). Consistent with its mechanism of action, inhibiting the late stage of viral replication, the antiviral activity of oseltamivir remained even when it was added at 8 h p.i. and a greater than 3 log_10_ units of viral titer reduction was observed. In contrast, hesperadin was found to inhibit the early stage of viral replication post viral entry and its antiviral efficacy gradually decreased when it was added at the later stages of viral replication (Figure 4B). Pre-treatment of cells with hesperadin from −24 h to −2 h or −12 h to −2 h had no effect on viral replication (Figure 3B). Similarly, hesperadin did not inhibit viral attachment and entry as shown by the results of the −2 to 0 h treatment. In summary, the results from the time-of-addition experiments suggest that hesperadin inhibits the early stage of viral replication post viral entry, possibly inhibiting viral RNA transcription and translation.

### 2.4. Hesperadin Inhibits Viral RNA Transcription, Translation and Viral Protein Synthesis

**Figure 5:**
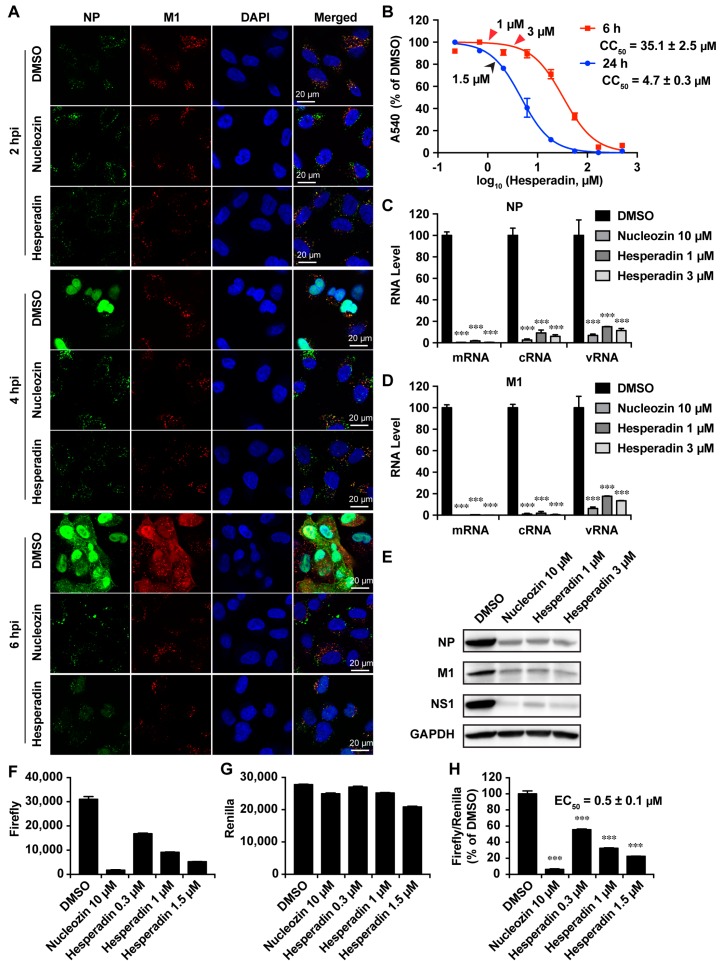
Hesperadin-inhibited viral replication via inhibition of vRNP nuclear entry. (**A**) Hesperadin delayed the vRNP nuclear entry. A549 cells infected with the A/WSN/33 (H1N1) virus (MOI = 30) were treated with DMSO, 10 µM of nucleozin or 1 µM of hesperadin. After fixation at indicated times (2, 4, and 6 h p.i.), cells were stained with mouse anti-influenza A NP antibody, rabbit anti-M1 antibody, and DAPI to determine the viral NP, M1 protein, and nucleus, respectively; (**B**) Cell viability of A549 cells when treated with hesperadin for 6 or 24 h. The arrows indicate the concentrations used (Red arrows: (**C**–**E**); Black arrows: (**F**–**H**). The CC_50_ value represent the average of eight repeats ± standard deviation; (**C**–**E**) Hesperadin reduced the levels of viral RNAs and proteins. A549 cells infected with the A/WSN/33 (H1N1) virus (MOI = 1) were treated with DMSO, 10 µM of nucleozin, 1 or 3 µM of hesperadin, respectively. At 6 h p.i., cells were harvested and total viral RNAs or proteins were extracted. The mRNA, cRNA and vRNA of NP and M1 were quantified by RT-qPCR (**C**,**D**); Viral protein NP, M1 and NS1 were examined by western blot (**E**); (**F**–**H**) Mini-genome assay. A549 cells were transfected with a combination of plasmids for mini-genome assay (see Materials and Methods). Hesperadin was added to the culture medium at 2 h post transfection. At 24 h post transfection, cells were harvested for measurement of Firefly (**F**) and Renilla (**G**) luciferase activities. Renilla served as an internal control to normalize the transfection efficacy (**H**). Asterisks indicate statistically significant difference in comparison with the DMSO control (student’s *t*-test, *** *p* < 0.001). The results represent the average of two repeats ± standard deviation.

To further dissect the mechanism of action of hesperadin we performed the immunofluorescence staining experiments (Figure 5A). A549 cells were infected with A/WSN/33 (H1N1) virus at a MOI of 30 and were treated with either DMSO or 1 µM hesperadin, which confers minimal cellular cytotoxicity (Figure 5B). The cells were fixed at 2, 4, and 6 h p.i. and stained with mouse anti-influenza NP antibody, rabbit anti-M1, and DAPI to determine the localization of the viral NP, M1 protein, and nucleus, respectively. Nucleozin, a known NP inhibitor, was included as a control [29]. It was found that nucleozin inhibited the nuclear entry of NP at both 4 and 6 h p.i. when compared to the DMSO control. Nucleozin also inhibited viral proteins NP and M1 synthesis as shown by the reduced fluorescence signals at 6 h p.i. when compared to the DMSO control. Similarly, treatment of hesperadin led to the delay of the nuclear entry of the NP protein, and the synthesis of viral proteins NP and M1 was also reduced at 6 h p.i. The immunofluorescence assay results suggest hesperadin might impair the viral polymerase activity by delaying the nuclear entry of the viral ribonucleoprotein (vRNP) complex. To test this hypothesis, we quantified the viral mRNA, cRNA, and vRNA levels by RT-qPCR at 6 h p.i. When treated with either 1 or 3 µM of hesperadin, the viral NP and M1 mRNA, cRNA, and vRNA levels were significantly reduced (Figure 5C,D). As a result, the expression levels of viral proteins NP, M1, and NS1 were also inhibited (Figure 5E). The inhibition of viral polymerase by hesperadin was further confirmed by the influenza mini-genome assay [30]. The mini-genome assay is the golden standard assay to test the function and inhibition of the viral polymerase complex. The EC_50_ of hesperadin in the minigenome assay is 0.5 ± 0.1 µM (Figure 5F–H), which is consistent with its antiviral efficacy (Table 1). Overall, immunofluorescence assay, RT-qPCR, western blotting, and mini-genome assay results collectively suggest hesperadin inhibits viral polymerase activity. As a result, viral RNA transcription, translation and protein synthesis are inhibited.

### 2.5. Hesperadin Shows Synergistic Antiviral Activity with Oseltamivir in Combination Therapy

As the antiviral mechanism of hesperadin is distinct to oseltamivir, we were interested in exploring the combination therapy potential of hesperadin with oseltamivir. The combination therapy potential was quantified by the fractional inhibitory concentration index (FICI) [31,32], with FICI less than, equal to, or greater than 1 indicating synergy, additivity, or antagonism, respectively [33]. The FICI values for all five sets of hesperadin and oseltamivir combination were less than 1 (Table 2), suggesting hesperadin has synergistic antiviral effect with oseltamivir in the combination therapy.

## 3. Discussion

Influenza viruses continue to represent a worldwide threat to public health, and the increasing resistance to current antiviral drugs presents a challenge to clinical health care. Therefore, newer antiviral agents with a novel mode of action are clearly needed. As viruses rely on cellular proteins for viral replication, identifying host factors that are involved in viral replication could provide promising new targets for antiviral therapy [15]. Moreover, host factors are less likely to develop mutations that make drug ineffective under certain drug selective pressure when compared to viral proteins. Furthermore, targeting host proteins provides potential broad-spectrum antiviral activity against various strains of influenza. Therefore, host-targeting antivirals represents a therapeutic strategy for influenza infection [34].

Recently, several studies using genome-wide screening have reported human genes or orthologs that are required for influenza viral replication [16,17,18,19,20,21,22]. Among the list of pro-viral host factors, kinases are one of the frequent hits [22,35]. This is probably because kinases are an essential aspect in many cellular processes, such as in signal transduction, inflammation, and immunity, rendering them high profile therapeutic targets [36,37,38]. Aurora B kinase is one of the three aurora family kinases involved in the regulation of cell division. It belongs to a family of serine/threonine kinases that are cell-cycle regulated and are activated during mitosis [39]. Hesperadin is an ATP-competitive inhibitor of aurora B kinase with IC_50_ of 250 nM [23]. It also inhibits other kinases such as AMPK, Lck, MKK1, MAPKAP-K1, CHK1, and PHK at 1 µM drug concentration. The cellular cytotoxicity of hesperadin in inhibiting HepG2 cells was reported to be 0.2 µM [40]. No in vivo anticancer activity of hesperadin has been reported. The antiviral activity of hesperadin ranges from 0.22 to 2.21 µM, which is in the same range as its in vitro kinase inhibition efficacy. This result suggests that aurora B kinase might be the antiviral drug target of hesperadin. Nevertheless, we cannot completely rule out the possibility that the antiviral effect of hesperadin might be due to its off-target effect(s) on other kinases.

As aurora B kinase is overexpressed in many cancer cells, aurora B kinase inhibitors such as hesperadin have been explored as anticancer agents [41]. However, the antiviral activity of hesperadin has not been reported. In this study, we identified hesperadin as a broad-spectrum influenza antiviral through the forward chemical genomics screening. Hesperadin inhibits multiple human clinic isolates of influenza A and B viruses at both low and high MOIs with a moderate to high selectivity index. Although the in vitro cellular selectivity index of hesperadin is not as high as direct-acting antivirals such as oseltamivir and amantadine, hesperadin might be well tolerated in vivo as influenza virus infection is an acute infection and drug treatment only needs to last for a week or two. It is noteworthy that at the therapeutic dose of inhibiting the influenza virus (1.5 µM, Figure 5G), hesperadin did not inhibit host protein synthesis as there was no reduction of the renilla luciferase signal. Nevertheless, the in vivo cytotoxicity of hesperadin needs to be validated by the maximum tolerated dose studies in mice or ferrets. The broad-spectrum antiviral activity of hesperadin suggests hesperadin might be a host-targeting antiviral. Mechanistic studies revealed that hesperadin delays the nuclear entry of viral ribonucleoprotein complex and inhibits viral polymerase activity in the mini-genome assay. This result suggests that aurora B kinase either directly or indirectly regulates the viral polymerase activity. As a result, viral RNA transcription, translation and viral protein synthesis are inhibited. Finally, the antiviral activity of hesperadin is synergistic with oseltamivir in combination therapy. Given the novel mechanism of action of hesperadin, it is worthwhile to further pursue hesperadin as an alternative influenza antiviral and use it as a chemical probe to delineate the role of aurora kinase B in the influenza virus replication cycle.

In summary, the discovery of hesperadin as a broad-spectrum influenza antiviral with a high in vitro genetic barrier to drug resistance is significant as it offers an alternative to combat emerging or re-emerging drug-resistant influenza strains. As hesperadin is still at the early stage of anticancer drug development, no animal result has been reported. Hence, to repurpose hesperadin as an influenza antiviral, follow up studies in mice or ferrets are urgently needed to further validate the safety and in vivo antiviral efficacy of hesperadin.

## 4. Materials and Methods

### 4.1. Cell Lines, Viruses, and Viral Infection

Madin-Darby Canine Kidney (MDCK) cells, genetically modified MDCK cells that overexpress ST6Gal I, and Adenocarcinomic human alveolar basal epithelial (A549) cells were maintained at 37 °C in in DMEM media (high glucose, with l-glutamine) supplemented with 10% fetal bovine serum (FBS), 100 U/mL penicillin, and 100 µg/mL streptomycin in a cell culture incubator with 5% CO_2_ atmosphere. MDCK cells overexpressing ST6Gal I [42] were kindly provided by Yoshihiro Kawaoka at the University of Wisconsin at Madison and were maintained in DMEM media with 7.5 µg/mL of puromycin, except when they were used for viral infection.

The following influenza A viruses, A/California/07/2009 (H1N1), A/Texas/04/2009 (H1N1), B/Brisbane/60/2008 (Victoria lineage), and B/Wisconsin/1/2010 (Yamagata lineage), were kindly provided by James Noah at the Southern Research Institute. Influenza A virus strain A/WSN/1933 (H1N1) was kindly provided by Robert Lamb at the Northwestern University. Influenza A virus strains A/Washington/29/2009 (H1N1) FR-460, A/North Carolina/39/2009 (H1N1) FR-488, A/Texas/12/2007 (H3N2) FR-1442, and A/California/02/2014 (H3N2) FR-1353, were obtained through the Influenza Reagent Resource, Influenza Division, WHO Collaborating Center for Surveillance, Epidemiology and Control of Influenza, Centers for Disease Control and Prevention, Atlanta, GA, USA. All viruses were amplified in MDCK cells with 2 µg/mL *N*-acetyl trypsin (NAT).

### 4.2. Plaque Assay

Antiviral plaque assay was performed using the MDCK cells expressing ST6Gal I according to the procedure described before [26,27].

### 4.3. Cytotoxicity Assay and Cytopathic Effect (CPE) Assay

CPE assay was used to evaluate the cytotoxicity as well as the antiviral efficacy of compounds [43]. Briefly, in each well of the 96-well cell culture plates, 100 µL of 80,000 cells/mL MDCK or A549 cells were suspended in DMEM medium that was supplemented with 10% FBS, 100 U/mL Penicillin, and 100 µg/mL streptomycin. After incubating for twenty-four hours to allow the cells to reach near confluence, the growth medium was removed by aspiration and the cells were washed by 100 µL PBS buffer. To determine the compound’s cellular cytotoxicity, 200 µL fresh DMEM (without FBS) medium containing serial half-log diluted compounds was added to each well. After incubating for 48 h with 5% CO_2_ in the cell culture incubator at 37 °C, the medium was removed and 100 µL DMEM medium containing 40 µg/mL neutral red was added. The solution was incubated for another 4 h at 37 °C in the cell culture incubator. The medium was removed and the amount of neutral red that was taken by the viable cells was dissolved by adding 100 µL of destaining solution (50% ethanol, 49% H_2_O, and 1% acetic acid). The absorbance of the solution at 540 nm was determined using a Multiskan FC Microplate Photometer (Thermo Fisher Scientific, Waltham, MA, USA). The CC_50_ values were plotted by the best-fit dose response curves with variable slope in Prism 5.

For CPE assay, cells at 8000 cells/well density were infected with 100 µL of 100 PFU influenza virus A/WSN/1933 (H1N1) for 1 h at 37 °C. Unabsorbed virus was removed and the cells were treated with various doses of tested compounds (0, 0.3, 1, 3, 10, 30, 100 µM). The uninfected cells and oseltamivir [25] positive control were included in each test. The plates were incubated at 37 °C for 44–48 h and neutral red was added when a significant cytopathic effect was observed in the wells without compound (virus only). The EC_50_ values were calculated from best-fit dose response curves with variable slope in Prism 5.

### 4.4. Time-of-Addition Experiment

A time-of-addition experiment was performed according to the procedure described earlier [28]. Briefly, MDCK cells were seeded at 6 cm^2^ dishes at a 2 × 10^5^ cells/dish cell density. After incubating for 24 h to allow cells to attach, the cells were infected with A/WSN/33 (H1N1) virus at a MOI of 0.01 at −2 h time point. Antiviral compounds, such as oseltamivir carboxylate (1 µM) or hesperidin (3 µM) was added at different time points before, during, or after viral infection as illustrated in Figure 4. Viruses were harvested from the cell culture supernatant at 12 h post infection. The virus titers were quantified by plaque assay.

### 4.5. Influenza Virus Minigenome Assay

A549 cells were seeded at a cell density of 3 × 10^5^ cells/well in 12-well plates and were incubated for 12 h at 37 °C in a CO_2_ cell culture incubator with 5% CO_2_. The cells were transfected with six plasmids which encode influenza A/WSN/33 (H1N1) virus PB1, PB2, and PA (100 ng each), NP (200 ng), the RNA polymerase II driven Renilla luciferase reporter pRL-SV40 (Promega, Madison, WI, USA) (250 ng), and the influenza virus specific RNA polymerase I driven firefly luciferase reporter (vRNA Luc) (250 ng). The transfection was achieved by the transfection reagent Lipofectamine 3000 (Thermo Fisher Scientific) in OptiMEM (Invitrogen, Carlsbad, CA, USA). Two hours after transfection compounds were added to the cell culture media. After incubating for twenty-four hours in the cell culture incubator, cells were harvested and lysed, and the expression of firefly luciferase and Renilla luciferase was quantified using the Dual Luciferase Assay Kit (Promega) according to the manufacturer’s protocol.

### 4.6. RNA Extraction and Real-Time PCR

Total RNA was extracted from influenza A/WSN/33 (H1N1) infected cells using Trizol reagents (Thermo Fisher Scientific). After removing genomic DNA by RQ1 RNase-Free DNase (Promega), the first strand of cDNA was synthesized using 1.2 μg of total RNA and AMV Reverse Transcriptase (Promega). vRNA specific primer (5′-AGCAAAAGCAGG-3′), cRNA specific primer (5′-AGTAGAAACAAGG-3′) or oligo (dT)18 was used for detecting influenza vRNA, cRNA or mRNA, respectively. Real-time PCR was performed on a StepOnePlus Real-Time PCR System (Thermo Fisher Scientific) using FastStart Universal SYBR Green Master (Rox) (Roche, Basel, Switzerland) and following influenza NP- or M1-specific primers: NP-F: 5′-AGGGTCAGTTGCTCACAAGTCC-3′; NP-R: 5′-TTTGAAGCAGTCTGAAAGGGTCTA-3′; M1-F: 5′-ATGGGAACGGAGATCCAAATAA-3′; M1-R: 5′-TGCACCAGCAGAATAACTGAGTG-3′. GAPDH was also amplified to serve as a control using human GAPDH-specific primers (GAPDH-F: 5′-ACACCCACTCCTCCACCTTTG-3′ and GAPDH-R: 5′-CACCACCCTGTTGCTGTAGCC-3′). The amplification conditions were: 95 °C for 10 min; 40 cycles of 15 s at 95 °C and 60 s at 60 °C. Melting curve analysis was performed to verify the specificity of each amplification. All experiments were repeated three times independently.

### 4.7. Western Blotting

Total proteins were extracted using RAPI lysis buffer (50 mM Tris pH 8.0, 1% NP-40, 0.1% SDS, 150 mM NaCl, 0.5% Sodium deoxycholate, 5 mM EDTA, 10 mM NaF, 10 mM NaPPi, 2 mM phenyl-methylsulfonyl, and 1 mM PMSF). Equal amount of extracted total proteins were separated by electrophoresis and transferred to a polyvinylidene difluoride (PVDF) membrane. Target protein was detected using the following antibodies and dilutions: rabbit anti-NP (GeneTex: GTX125989) at 1:5000, rabbit anti-M1 (GeneTex: GTX125928) at 1:5000, rabbit anti-NS1 (GeneTex: GTX125990) at 1:5000, mouse anti-GAPDH antibody (EMD Millipore: MAB374) at 1:3000; detection used horse radish peroxidase (HRP)-conjugated secondary antibodies at 1:3000 and Supersignal West Femto substrate (Thermo Fisher Scientific, Waltham, MA, USA).

### 4.8. Immunostaining

Influenza A/WSN/33 (H1N1) infected cells were fixed with 4% formaldehyde for 10 min followed by permeabilization with 0.2% Triton X-100 for another 10 min. After blocking with 10% bovine serum, cells were stained with mouse anti-NP (1:200 dilution; Bio-Rad: MCA400, Hercules, CA, USA) and rabbit anti-M1 (1:200 dilution; Gene Tex: GTX125928) antibodies, followed by incubation with anti-mouse secondary antibody conjugated to Alexa-488 and anti-rabbit secondary antibody conjugated to Alexa-594 (Thermo Fisher Scientific, Waltham, MA, USA). Nuclei were stained with 300 nM DAPI (Thermo Scientific) after secondary antibody incubation. Fluorescent images were acquired using a ZOE Fluorescent Cell Imager (Bio-Rad).

### 4.9. Assessment of Combination Treatment of Hesperadin with Oseltamivir In Vitro

The synergistic antiviral effect of hesperidin and oseltamivir carboxylate was evaluated in cell cultures as described previously [44] using CPE assay as described above in Section 4.3, except that the compounds added were oseltamivir carboxylate or hesperadin, alone or in combinations. Five combinations of hesperadin and oseltamivir, at the fixed EC_50_ ratios of 10:1, 5:1, 1:1, 1:5 and 1:10, were included. In each combination, a stock solution with the designated EC_50_ ratio of hesperidin and oseltamivir was first made, then six 3-fold serial dilutions (equal to 0.5 log_10_ unit decrease) of the stock solution were tested to obtain the dose inhibition curve, based on which EC_50_ of individual hesperadin or oseltamivir was determined. Subsequently, fractional inhibitory concentration index (FICI) was calculated using the following formula: FICI = ((EC_50_ of hesperadin in combination)/(EC_50_ of hesperadin alone)) + ((EC_50_ of oseltamivir in combination)/(EC_50_ of oseltamivir alone)). FICI < 0.5 was interpreted as a significant synergistic antiviral effect [45].

## Figures and Tables

**Table 1:** EC_50_ of hesperadin against a panel of influenza A and B viruses.

Influenza Strains	Drug Sensitivity	EC_50_ (µM) ^a^	SI ^b^
A/Texas/4/2009 (H1N1)		1.07 ± 0.11	19.9
A/North Carolina/39/2009 (H1N1)		0.82 ± 0.02	26
A/Washington/29/2009 (H1N1)	Amantadine Resistant	0.37 ± 0.12	57.6
A/California/02/2014 (H3N2)	Oseltamivir Resistant	1.80 ± 0.42	11.8
A/Texas/12/2007 (H3N2)		2.21 ± 0.23	9.7
A/WSN/1933 (H1N1)	Amantadine Resistant	0.22 ± 0.04	56.1
A/California/07/2009 (H1N1)	Oseltamivir Sensitive	0.28 ± 0.03	76.1
B/Wisconsin/1/2010 (Yamagata)	Amantadine Resistant	0.73 ± 0.05	29.2
B/Brisbane/60/2008 (Victoria)	Oseltamivir Sensitive	0.52 ± 0.05	41

^a^ All EC_50_ results were determined in plaque assays in duplicates using the ST6Gal I-overexpressing MDCK cells (AX-4) [26] in 6-well plates; The EC_50_ value represents the average of two repeats ± standard deviation; ^b^ SI refers to the selectivity index, which is calculated by dividing the 48 h CC_50_ of hesperadin by the EC_50_ of hesperadin.

**Table 2:** Antiviral results of combinational treatments.

Combination Ratio (EC_50_)	EC_50_ in Combination		EC_50_ Alone		EC_50_ Equivalent ^a^		FICI ^b^
Hesperadin: Oseltamivir	Hesperadin (μM)	Oseltaimivir (nM)	Hesperadin (μM)	Oseltaimivir (nM)	Hesperadin	Oseltaimivir	
10:1	0.48 ± 0.15	1.1 ± 0.25			0.238	0.106	0.34
5:1	0.56 ± 0.05	2.26 ± 0.20			0.277	0.217	0.49
1:1	0.17 ± 0.20	3.44 ± 1.01	2.02 ± 0.18	10.10 ± 3.11	0.084	0.331	0.41
1:5	0.037 ± 0.006	3.73 ± 0.62			0.018	0.359	0.38
1:10	0.034 ± 0.052	6.28 ± 1.17			0.017	0.604	0.62

Values are means ± SD (*n* = 8) from two independent experiments. ^a^ Concentration in EC_50_ equivalent was the normalized concentration that was calculated by dividing the EC_50_ of drug in combination with its EC_50_ alone; ^b^ FICI was the sum of hesperadin and Oseltamivir EC_50_-equivalent concentrations used in each combination.

## Abbreviations

HA	Hemagglutinin
NA	Neuraminidase
MOI	Multiplicity of infection
CPE	Cytopathic effect
vRNP	Viral ribonucleoprotein
RT-qPCR	Real-time quantitative polymerase chain reaction
FICI	Fractional inhibitory concentration index
MDCK	Madin–Darby canine kidney
